# Interpreting Prostate MRI Reports in the Era of Increasing Prostate MRI Utilization: A Urologist’s Perspective

**DOI:** 10.3390/diagnostics14101060

**Published:** 2024-05-20

**Authors:** Kevin Miszewski, Katarzyna Skrobisz, Laura Miszewska, Marcin Matuszewski

**Affiliations:** 1 Department of Urology, Gdańsk Medical University, Mariana Smoluchowskiego 17 Street, 80-214 Gdańsk, Poland; 2 Department of Radiology, Gdańsk Medical University, Mariana Smoluchowskiego 17 Street, 80-214 Gdańsk, Poland; 3 Student Scientific Association, Gdańsk Medical University, Mariana Smoluchowskiego 17 Street, 80-214 Gdańsk, Poland

**Keywords:** prostate cancer, MRI, standardized reporting, prostate biopsy

## Abstract

Multi-parametric prostate MRI (mpMRI) is crucial for diagnosing, staging, and assessing treatment response in individuals with prostate cancer. Radiologists, through an accurate and standardized interpretation of mpMRI, stratify patients who may benefit from more invasive treatment or exclude patients who may be harmed by overtreatment. The integration of prostate MRI into the diagnostic pathway is anticipated to generate a substantial surge in the demand for high-quality mpMRI, estimated at approximately two million additional prostate MRI scans annually in Europe. In this review we examine the immediate impact on healthcare, particularly focusing on the workload and evolving roles of radiologists and urologists tasked with the interpretation of these reports and consequential decisions regarding prostate biopsies. We investigate important questions that influence how prostate MRI reports are handled. The discussion aims to provide insights into the collaboration needed for effective reporting.

## 1. Introduction

Prostate cancer (PCa) is one of the most prevalent malignancies affecting men worldwide, with a significant impact on morbidity and mortality [1]. In the quest for more accurate diagnostic and prognostic tools, multiparametric magnetic resonance imaging (mpMRI) has emerged as a valuable asset in urology practice [2]. Multiple trials revealed the great potential of mpMRI for performing pre-biopsy diagnosis [3]. In the era before MRI became a standard practice, random systematic biopsies were associated with alarmingly high false-negative rates, reaching up to 76% [3]. The introduction of MRI represents a substantial shift, offering patients a valuable tool to steer clear of the potentially severe consequences associated with biopsies and improving the detection rates of clinically significant prostate cancer (CSPCa) and lower rates of detection of clinically insignificant cancer [4]. As a result, the latest 2023 European Association of Urology (EAU) guidelines recommend prostate MRI in asymptomatic men with PSA 3–10 ng/mL and normal DRE [2]. However, incorporation of prostate MRI in the diagnostic pathway will lead to an increase in demand for high-quality mpMRI in Europe and the USA [5,6]. This is predicted to equate annually to approximately two million additional prostate MRI scans [7]. Research conducted by Davies et al. [8] in 2019 has indicated that multiparametric MRI (mpMRI) availability surpasses 90% across diverse regions in the United Kingdom, highlighting the extensive accessibility of MRI services despite certain regional variances. Nevertheless, the status of accessibility in less developed countries remains uncertain. The current widespread utilization of pre-biopsy prostate MRI has a significant impact on healthcare, specifically in its immediate effects on the workload and roles of radiologists and urologists responsible for interpreting these reports and making decisions about prostate biopsies. Interpreting prostate MRI images requires a specialized skill set. As the number of scans increases, the demand for radiologists with expertise in prostate MRI interpretation also grows. Ensuring a sufficient number of qualified radiologists is essential to maintain the accuracy and reliability of prostate MRI reports. A comprehensive array of reporting and data standards, with a specific emphasis on cancer imaging, has been meticulously developed, organized, and overseen by the American College of Radiology (ACR) [9]. These standards, collectively known as RADS, encompass well-known frameworks such as PI-RADS, BI-RADS, LI-RADS, and numerous others. It is crucial to recognize that all these RADS serve as dynamic resources undergoing continuous updates with new versions regularly released to ensure their relevance and applicability in the rapidly evolving field of medical imaging [10]. The initial effort to standardize prostate MRI reporting commenced with the release of the Prostate Imaging Reporting and Data System (PI-RADS) guidelines version 1 in 2012 [10]. These guidelines delineated the essential technical prerequisites and standard criteria for reporting prostate mpMRI findings. As evidence accumulated through their widespread use, the PI-RADS guidelines underwent subsequent refinements in 2015 (version 2.0) and further improvements in 2019 (version 2.1) [11,12]. These introduced a five-point assessment scale to assess the probability of a correlation between the findings obtained from mpMRI and the presence of CSPCa at a specific anatomical site. Prior research has confirmed the effectiveness of positive mpMRI results in detecting CSPCa [13,14] Due to its widespread use and acknowledged utility, the Prostate MRI Quality Subcommittees of the European Society of Urogenital Radiology (ESUR) and the European Association of Urology Section of Urologic Imaging (ESUI) formulated consensus-based criteria for prostate MRI acquisition, reporting, and training [15]. However, the practical implementation of PIRADS reporting in routine clinical practice presents a multifaceted challenge, encompassing interpretative complexity, interobserver variability [16,17], and the need for continuous training and refinement. In this era of precision medicine, where tailored treatments are becoming the norm, the dialog between radiologists and urologists must evolve to meet the demands of a rapidly advancing field. The goal is clear: to ensure that every patient receives the most accurate diagnosis, appropriate treatment, and the best possible outcome. We investigate the key questions that shape the landscape of prostate MRI reporting and highlight the crucial role played by the radiologist–urologist partnership. The discussion encompasses the complexities of prostate MRI reporting in practice, striving to provide insights into optimal practices for accurate and comprehensive reporting.

## 2. Should MRI Reports Be Structured or Presented in Free-Text Format?

Traditionally, radiologists have favored the expressive flexibility of free-text reporting, allowing them to articulate nuanced observations and individualized insights. However, the inherent complexity and subtle nature of prostate imaging necessitate a meticulous and standardized approach to reporting, making structured reporting (SR) an attractive proposition for streamlining the communication of diagnostic information. In the Magnetta [18] paper, it was demonstrated that after implementing SR, improvements in consistency, completeness, clarity, and clinical impact of the reports were observed, alongside a reduced perceived need to contact the interpreting radiologist for further clarification. Furthermore, structured reporting templates improved the sensitivity of prostate MRI for CSPCa in the peripheral zone from 53 to 70% [19] Faggioni et al.’s [20] survey findings indicate that the implementation of radiological SR offers distinct advantages over conventional reporting. Noteworthy strengths identified by respondents encompass heightened report reproducibility, enhanced communication channels between radiologists and referring clinicians, and the facilitation of more concise reports. However, the survey results reveal a striking trend, indicating that radiological SR is either not utilized at all or adopted by less than 50% of the radiological staff in many centers. This underutilization implies a de facto reluctance among radiologists to transition from conventional reporting to the adoption of SR in their daily practice. This hesitancy may be attributed to perceived disadvantages and current limitations associated with radiological SR. Respondents highlighted two main weaknesses: the risk of excessive report simplification in complex cases and the perceived rigidity of reporting templates. These concerns contribute to the prevailing resistance towards embracing SR in routine radiological reporting. The perspective of urologists underscores the critical importance of standardized reporting. Beyond the imperative of diagnostic accuracy, clinicians place a premium on linguistic clarity in radiology reports [21,22]. Extensive research has consistently demonstrated that urologists prefer SR within the PIRADS framework [23,24]. The evidence presented here highlights the potential for structured reports to not only streamline reporting practices but also contribute to better patient outcomes, reduced variability in reporting, and improved training for new radiologists. Furthermore, the structured format enables data extraction for research purposes, which can support ongoing clinical studies and quality improvement initiatives [25].

## 3. How Many Lesions Should Ideally Be Described within a PI-RADS Report?

Urologists, tasked with interpreting and utilizing PIRADS reports for clinical decision-making, often find themselves navigating through a multitude of lesions, a scenario that can inadvertently lead to decision fatigue. The sheer volume of lesions, coupled with the inclusion of those with lower clinical significance, may compromise the precision and efficiency of decision-making processes. In the PI-RADS 2.1 paper [26], comprehensive guidelines have been delineated for the structured reporting of lesions. According to these guidelines, a maximum of four lesions, each carrying a PI-RADS assessment score of 3, 4, or 5, can be assigned within each sector map. Addressing scenarios where the total number of lesions exceeds four, the reporting process is refined to encompass only the four lesions displaying the highest likelihood of CSPCa. In some quarters, the consensus suggests that a PI-RADS report should typically encompass a maximum of three lesions, reflecting a pragmatic approach to clinical decision-making. Such an approach aligns with the belief that an excessive enumeration of lesions may introduce complexity into the interpretation process, potentially overwhelming clinicians and impeding the identification of CSPCa. In their research, Spilseth et al. [23] found that radiologists and urologists most frequently indicated that three lesions are the maximum number of lesions that should be reported, though, surprisingly, urologists were more likely than radiologists to indicate that five or more lesions should be included. However, it is essential to acknowledge that the medical community is not unequivocal in its stance on this matter. Within the societies of urologists and radiologists, diverse opinions and practices prevail. Some advocate for a more inclusive approach, contending that a comprehensive enumeration of all detectable lesions, regardless of quantity, may provide valuable information for patient management and follow-up.

## 4. Is It Appropriate for Radiologists to Utilize Terms Such as “PIRADS 3/4” in Their Reports Even When These Specific PIRADS Scores Are Not Explicitly Designated?

While such terminology might offer a degree of flexibility in reporting, it simultaneously poses challenges in terms of diagnostic precision. The ambiguity inherent in these combined scores can complicate the decision-making process for prostate biopsies, potentially leading to under- or overdiagnosis of clinically significant lesions. Within the spectrum of PI-RADS scores, a significant divide emerges between PI-RADS 3 and PI-RADS 4 findings, leading to distinct clinical implications. PI-RADS 3 represents a category recognized for its ongoing debate in clinical practice, largely due to its association with a higher rate of false-positive results in prostate biopsies [27]. In the MRI-FIRST, PRECISION, and 4M trials, biopsy-naïve patients with PI-RADS 3 lesions exhibited CSPCa of 7–15% when undergoing targeted biopsies [28,29,30]. In the FUTURE trial, biopsy-naïve patients with PI-RADS 3 lesions had a CSPCa of 8.7% when undergoing targeted biopsies [31]. On the contrary, PI-RADS 4 is a designation that raises heightened concern, as it signifies a substantial likelihood of harboring a neoplasm or malignancy within the prostate gland. Westphalen et al. [32], who evaluated the positive predictive value of MRI-directed biopsy for detection of CSPca in 3449 men with positive MRI scans showed positive predictive value for CSPCa detection of 49% (95% CI 40–58%, IQR 27–48%) for PI-RADS 4. These studies demonstrate a significant distinction between PIRADS 3 and 4. However, the radiology community faces a substantial challenge. The utilization of vague terms like PIRADS 3/4 is not their fault; the PIRADS system lacks the flexibility to effectively describe various lesions, particularly those in the transitional zone. In the paper authored by Messina [33], the issue of ambiguous PI-RADS reporting was tackled by introducing a novel subcategorization of PI-RADS 3 scoring. This new subcategorization comprises PI-RADS 3 and 3up findings, which are further divided into two distinct groups: PI-RADS 3B, which necessitates immediate biopsy based on clinical data; and PI-RADS 3FU, indicating the need for follow-up with additional MRI assessments. A different approach to addressing this challenge has arisen with the development of computer-aided diagnosis (CAD) systems. Ferierro et al. [34]. highlighted the utility of these systems, revealing that radiologists acknowledged the advantages of computational analysis in approximately 15.3% of cases where the PI-RADS Score was ≤3. In the majority of these instances, T2 hypointensity was indistinct, yet a positive Malignancy Attention index map provided by the CAD system assisted radiologists in distinguishing suspicious foci from benign stromal nodules.

In conclusion, it is evident that the judicious use of standardized PIRADS scores and clear, precise reporting practices remains paramount in prostate MRI reporting. While the temptation to employ vague terms may exist, the potential risks associated with such practices underscore the importance of adhering to established reporting guidelines. In cases where uncertainty or challenges arise in adhering strictly to the PIRADS criteria, the importance of engaging in multidisciplinary team discussions and fostering close collaborative relationships between radiology and urology cannot be overstated.

## 5. Should Radiologists Routinely Incorporate TNM (Tumor, Node, Metastasis) Staging Criteria in Their Reports?

The TNM staging system for PCa, originally introduced in 1992 [35], holds a pivotal role in precisely characterizing the overall cancer burden, assessing the extent of disease spread at the point of diagnosis, and categorizing patients into prognostic groups. The present method for preoperative risk assessment in PCa relies on nomograms like the D’Amico criteria, Partin tables, or the EAU risk groups. These tools were originally formulated and validated based on the clinical stage determined through digital rectal examination (DRE) [36,37,38]. However, research indicates a significant discordance between clinical DRE staging and the final pathological findings. In a study conducted by Philip et al. involving 408 men, it was revealed that DRE exhibited a 60% under-staging rate for individuals with a histological diagnosis of cancer. Remarkably, nearly 40% of patients initially categorized as having a normal DRE (T1c) were ultimately classified as T2 or T3 in the final pathology [39]. Another investigation demonstrated a substantial 70% upstaging from clinical T2a disease to T2c disease upon examination of final pathology [40]. Furthermore, DRE exhibited poor correlation in accurately delineating the location and extent of the disease. These findings carry significant clinical implications as the current guidelines for nerve-sparing prostatectomy, as outlined by the EAU, rely on indications and contraindications derived from trials where patient selection was predominantly informed by DRE staging [41]. Nerve-sparing surgery is generally restricted to patients with organ-confined disease. Extension of PCa outside the prostatic capsule requires dissection of the neurovascular bundle, for nerve-sparing surgery would increase the risk of positive surgical margins [41]. In contrast to EAU guidelines, MRI-based staging altered the eligibility for nerve-sparing prostatectomy in 27% of cases, leading to a shift towards less nerve-sparing surgery in the majority [42]. This highlights the crucial role of MRI in refining treatment decisions and underscores the potential for more intensified treatment in about 1 out of 4 patients when relying on MRI rather than DRE for clinical tumor staging. However, the low specificity of mp-MRI for the detection of stage ≥T3a tumors, and thus its increased risk of over-staging, shows the technique is still not perfect [43]. Extraprostatic extension (T3a), invasion into seminal vesicles (T3b), and infiltration into neighboring structures (T4) are associated with a less favorable prognosis. They also increase the risk of positive surgical margins and biochemical recurrence following initial therapy [44]. A 2016 meta-analysis of 75 total studies found that pooled data for Extraprostatic Extension (EPE) showed sensitivity and specificity of 57% and 91%, respectively, and pooled sensitivity and specificity for SVI (seminal vesicle involvement) were 58% and 96%, respectively, concluding that MRI has high specificity but poor sensitivity for local PCa staging [45]. Recognizing the local extent of disease advancement is crucial as it can influence the scope of surgical intervention, the efficacy of surgical treatment, and the evaluation of alternative therapeutic options. Druskin et al. noted the presence of positive surgical margins in areas identified by preoperative MRI as indicative of extracapsular extension (ECE). These observations imply that urologists should be mindful of the potential necessity for a broader resection in cases where preoperative MRIs suggest locally advanced disease [46]. In a study conducted by Haug et al. [47], the implementation of pre-biopsy MRI was demonstrated to influence treatment decisions in PCa in Norway. This resulted in a decline in the number of locally advanced high-risk patients undergoing surgery, with a preference for radiotherapy. Additionally, a significant reduction in positive surgical margins for pT3 tumors was observed during the same period. These findings suggest an improvement in patient selection between radiotherapy and surgery and enhanced treatment planning, particularly for patients with Gleason Grade (GG) ≥ 3 [48]. Continual endeavors are underway to advance the staging methods for PCa. For instance, there has been a recent publication that specifically centers around the adaptation of the EAU risk group models to integrate mpMRI alongside DRE. This integrated model demonstrates promising prospects for refined risk stratification, exhibiting a heightened precision in predicting progression compared to the conventional EAU risk group classification [48].

These findings substantiate the notion that the incorporation of prostate MRI reports alongside TNM staging offers valuable staging insights that can significantly contribute to informed clinical decision-making. In accordance with Zhang’s [24] research, it was observed that urologists displayed a higher degree of satisfaction with reports incorporating TNM staging as opposed to reports containing solely PIRADS information.

## 6. Is There a Significant Impact on the Diagnostic Accuracy When Radiologists Include a Sector Map of the Prostate in a PIRADS Report?

Historically, radiologists have relied on subjective descriptions or general anatomical regions to denote the location of abnormalities within the prostate. This conventional approach, often described using vague terms such as “right posterior peripheral zone” or “left mid-gland”, lacks precision. In recent years, a growing body of evidence underscores the value of sector mapping in MRI reports for PCa assessment. PI-RADS v2 introduced a 39-sector prostate mapping system, comprising 36 sectors designated for the prostate, 2 for the seminal vesicles, and 1 for the external urethral sphincter. It was designed to enhance precision in localizing targeted biopsy procedures [11,49]. PI-RADS v2.1 has introduced two additional regions located in the peripheral zone (PZ) at the level of the base: the right and left posterior PZ medial. This brings the total number of sectors to 41. In contrast to the recommendations set forth by PI-RADS v.2, it is noteworthy that radiologists generally exhibit a notable reluctance towards the utilization of a sector map as the preferred method for lesion localization in clinical practice [50]. However, amidst the excitement surrounding mp-MRI, a crucial aspect often finds itself relegated to the periphery of radiological discourse—the cognitive prostate biopsy. Cognitive prostate biopsy remains an indispensable tool in the armamentarium of urologists for PCa diagnosis. Despite the emergence of more advanced techniques, such as fusion biopsy, cognitive biopsy maintains its significance primarily due to its widespread availability and accessibility. While fusion biopsy offers enhanced precision through the fusion of mpMRI with real-time ultrasound guidance, cognitive biopsy remains an essential option, especially in settings where access to specialized equipment or expertise may be limited. Urologists can readily perform cognitive biopsies using conventional transrectal ultrasound (TRUS) guidance and sector maps without the need for dedicated fusion platforms. The current literature, including systematic reviews and meta-analyses, does not show a clear superiority of one image-guided technique over another [31]. Arsov et al. [51] discovered that there was no noteworthy distinction in the detection of both overall PCa (37% vs. 39%) and CSPCa (29% vs. 32%) between in-bore MRI biopsy and MRI-TRUS fusion biopsy. Yaxley et al. [52] similarly observed no superiority of in-bore MRI biopsy over cognitive TRUS biopsy in identifying overall PCa and CSPCa. In a prospective trial conducted by Hamid et al. [53], there were no significant differences in the rates of overall PCa and CSPCa detection between cognitive and MRI-TRUS fusion techniques.

In conclusion, while fusion biopsy techniques continue to gain traction and offer valuable insights in PCa diagnosis, cognitive biopsy remains an indispensable and resilient methodology. Moreover, cognitive biopsy remains a feasible alternative in situations where advanced imaging resources may be scarce. It is noteworthy that radiologists frequently conduct prostate biopsies and, due to their proficiency in reading MR images, are less reliant on a sector map.

## 7. To What Degree Does the Inclusion of PSA Density and Prostate Volume Enhance the Clinical Utility and Precision of a PIRADS Report?

Prostate-specific antigen (PSA) has long been a cornerstone in the early detection of PCa, serving as a valuable biomarker for assessing disease risk. However, the traditional use of PSA levels alone as a sole criterion for recommending prostate biopsy has been a subject of debate due to its limitations, including false positives and the potential for overdiagnosis [54]. In recent years, PSA density has emerged as a promising adjunctive tool that offers a more refined and personalized approach to patient qualification for prostate biopsy [55]. Prostate-specific antigen density, calculated as the PSA level divided by the prostate volume (PV) as determined by imaging (usually transrectal ultrasound or MRI), provides a more complex understanding of PSA dynamics within the context of the individual patient’s prostate size. This metric addresses a fundamental limitation of using PSA levels alone, as it accounts for variations in prostate size that can significantly affect PSA concentration. One of the key advantages of incorporating PSA density into the qualification process for prostate biopsy is its ability to reduce unnecessary biopsies among patients with elevated PSA levels but smaller prostates. The IMRIE study [56], which retrospectively examined 2642 men, revealed that incorporating the standard PSAd (≥0.15 ng/mL/cc) into the MRI-pathway resulted in increased sensitivity and negative predictive value (NPV) for Gleason Grade (GG) ≥ 2 (87.3–96.6% and 87.5–90.6%). Furthermore, the utilization of a PSA density of 0.12 ng/mL^2^ further improved sensitivity and NPV in this context. In a patient-centered analysis [57], the detection of CSPCa was enhanced by 7% for PI-RADS 3, 17% for PI-RADS 4, and 15% for PI-RADS 5 when employing a PSAd cutoff of ≥0.1 ng/mL/cc. In a study conducted by Vourganti et al., no high-grade cancers were detected in patients with a PSA density of less than 0.15 ng/mL^2^. Furthermore, the research revealed that only PSA density (with a significance level of *p* = 0.0026) and MRI suspicion level (with a significance level of *p* = 0.0334) were notable factors for predicting the outcomes of biopsies [58]. Nevertheless, these assessments cannot be formulated without the measurement of PV. Ultrasonography, exemplified by techniques such as Transabdominal Ultrasound (TAUS) and Transrectal Ultrasonography (TRUS), is frequently employed in clinical settings. Notably, TRUS stands out as the predominant modality, commonly utilized as the primary tool for estimating PV [59]. MRI has also been widely adopted for the assessment of PV [60], owing to its established precision in measurement, making it the most accurate method in this regard [61,62]. PI-RADS v2.1 has delineated guidelines to ensure a consistent and systematic method of calculating PV in the ellipsoid formulation. PI-RADS v2.1 recommends that both maximum AP and longitudinal diameters be placed on the mid-sagittal T2W image and that the maximum transverse diameter be placed on the axial T2W image to optimize accuracy in the measurement of PV when using the ellipsoid formulation [12]. Evidence suggests that MRI, characterized by superior precision, can significantly impact PSA density calculations made during outpatient visits, consequently altering risk categorization and influencing the choice of biopsy.

The studies mentioned above unequivocally illustrate the valuable contribution of PSA density and PV when employed alongside PI-RADS in guiding clinical decision-making. Its primary usefulness appears to be in the context of pre-biopsy evaluations, where men identified as having a low probability of CSPCa based on their PI-RADS score and PSA density may be spared unnecessary biopsies. The incorporation of PSA density into the interpretation of prostate MRI findings is a logical progression towards improving diagnostic precision. In addition to risk stratification, PV plays a crucial role in determining the optimal number of needle cores needed for an effective non-targeted prostate biopsy [63]. Therefore, it is imperative for radiologists to consistently include PSA density based on MRI measurements of the prostate in their reports. Nevertheless, incorporating PSA density into MR reports may face challenges, as some MR protocols lack a sagittal sequence necessary for calculating PV, and not all requests include the PSA level.

## 8. Should Radiologists Actively Engage in Advising Urologists about the Necessity of Prostate Biopsy Based on Their Interpretations of PI-RADS Reports?

While radiologists possess specialized expertise in interpreting imaging data, the urology community may express reservations regarding such active advising, primarily due to their extensive clinical knowledge and experience in the field of prostate health. Urologists often emphasize in surveys the necessity of indicating the percentage likelihood that a lesion represents cancer and specifying whether a lesion is suitable for targeted biopsy [23]. Within the PIRADS 2.1 framework, the term “foremost lesion” has been introduced to designate the index lesion, emphasizing the imperative of its unequivocal identification [12]. The index lesion is determined based on the highest PI-RADS score. In instances where two or more lesions share an identical highest score, priority is accorded to the lesion demonstrating EPE. Notably, if a smaller lesion exhibits EPE, even in the presence of larger lesions, it is specifically designated as the index lesion. In the absence of EPE among lesions with the same highest score, the index lesion is established as the largest one [26]. This information can serve as an additional piece of the diagnostic puzzle, potentially aiding urologists in making more informed decisions. Radiologists’ insights regarding lesion characteristics, size, and location can provide valuable context for urologists to consider when determining whether a prostate biopsy is necessary or which technique of biopsy to choose; for example, transperineal fusion biopsy for lesions in the apex of the prostate is often unavailable for systemic biopsy.

## 9. Conclusions

To bridge the gap between two perspectives, it is essential for radiologists and urologists to establish effective communication channels and collaborative protocols. Open dialog and mutual respect for each other’s expertise can lead to a more comprehensive and patient-centered approach to PCa diagnosis and treatment decisions.

## Data Availability

Data sharing is not applicable to this article as no new data were created or analyzed in this study. All data discussed in this review are from previously published sources, which are appropriately cited within the paper.
